# Expression of sex hormone-binding globulin gene and its relation to serum testosterone concentration in *Bubalus* buffaloes

**DOI:** 10.1007/s11250-025-04592-4

**Published:** 2025-08-05

**Authors:** Samy Naeem, Ahmed M. Ghoneim, Hanan Tag-Eldin

**Affiliations:** 1 Animal Health Research Institute (Damietta Lab), Agriculture Research Center (ARC), Giza, 12618 Egypt; 2 Department of Zoology, Faculty of Science, Damietta University, P.O. 34517, New Damietta, Egypt; 3 Hormones Unit, Chemistry Department, Agriculture Research Center (ARC), Animal Health Research Institute, Dokki, Giza, 12618 Egypt

**Keywords:** Buffaloes, Testosterone, SHBG, Gene expression, Promoter, Polymorphism

## Abstract

To be bioavailable and exert its roles, testosterone binds to the sex hormone-binding globulin (SHBG) synthesized mainly by the hepatic cells. We investigated the relation between blood testosterone, SHBG gene expression in the hepatic cells, and the genetic variations in SHBG gene on Egyptian Bubalus bubalis buffaloes. Serum testosterone concentration increased significantly with age up to 36 months, then slightly declined at 42 months. *SHBG mRNA* levels slightly fluctuated in the different ages and declined at 42 months. Levels of SHBG protein were higher at 30 months, declined gradually with age up to 36 months, then increased slightly at 42 months. Serum testosterone concentration positively correlated with male buffaloes’ age. SHBG protein level negatively correlated with male buffaloes’ age and serum testosterone concentration. The positions −703 and −674 of SHBG gene promoter were polymorphic and the allele ‒674T could be associated with increased transcription of SHBG gene. A 603 bp-region in the middle of SHBG gene was determined as highly polymorphic; with 3–10 polymorphic sites. Certain genotypes were associated with higher or lower serum testosterone concentration. These results are important for characterizing the genetic make-up of SHBG gene, which may facilitate identifying high- concentration testosterone bull genotypes.

**Supplementary Information:**

The online version contains supplementary material available at 10.1007/s11250-025-04592-4.

## Introduction

The buffalo population in the world is about 204 million heads, with 98% of which distributed in Asia and the remainder is spread in Africa, mostly in Egypt, South America, Australia, and Europe (FAO 2022). Testosterone is a steroid hormone that is found in mammals, reptiles (Reisch et al. 2010), and birds (George et al. 2013). Some studies have been directed to estimate the testosterone quantities in buffalo bulls and to explore the effect of age on its concentration (Naeem et al. 2018; Gulia et al. 2010; Ahmad et al. 1984, 1989; Hemeida et al. 1985).

Sex hormone-binding globulin (SHBG) is synthesized as a homodimeric glycoprotein and released into the circulation mainly by the hepatic cells (Hammond 2011). SHBG binds biologically active sex hormones with high affinity (mainly 5-alpha-dihydrotestosterone, testosterone, and 17-beta-estradiol) and controls their availability for tissues under effect (Dunn et al. 1981; Li et al. 2016; Tint et al. 2016). Androgen-binding protein (ABP) is an SHBG homologue manufactured by Sertoli cells in mammalian tests (Joseph 1994). SHBG and ABP proteins are encoded by the same functional gene (Gershagen et al. 1989).

The quantities of human SHBG and testosterone in blood are believed to be controlled by some genetic causes (Ahn et al. 2009; Ohlsson et al. 2011; Chen et al. 2013; Sung and Song 2016). These studies identified some single-nucleotide polymorphisms (SNPs) in human SHBG gene plus some other genes that were significantly associated with SHBG and serum testosterone concentrations. Based on screening the sequence of SHBG gene, Naeem et al. (2018) noticed a potential association between either lower or higher serum testosterone concentration and some genotypes.

SHBG transcripts were studied in different tissues of humans and many other species. Human SHBG/ABP mRNA transcripts were detected in liver and many other organs. SHBG mRNA was found in rabbit liver and testis (Wong et al. 2001), kidney and brain (Ng et al. 2005). Also, SHBG mRNA was expressed in rat hypothalamus (Sendemir et al. 2006) and dog testes (Dalmazzo et al. 2019) and in the hepato-pancreas and other organs of pejerrey fish (González et al. 2017). Two transcript isoforms, SHBG-β and SHBG-α, were detected in different organs of adult rainbow trout (Bobe et al. 2008) and salmon pre-smolts (Miguel-Queralt et al. 2009). SHBG protein was discovered in the liver and different organs of the adult zebrafish and sea bass (Miguel-Queralt et al. 2004, 2007; Miguel-Queralt and Hammond 2008).

Human SHBG gene expression is modulated by a couple of transcription factors including hepatocyte nuclear factor 4 alpha (HNF-4α), constitutive androstane receptor, peroxisome proliferator-activated receptor gamma (PPAR-2γ) and chicken ovalbumin upstream promoter transcription factor1 (COUP-TF1) (Jänne and Hammond 1998; Saez-Lopez et al. 2017; Selva and Hammond 2009a). These transcription factors, in turn, are influenced by hormonal, metabolic, nutritional, and inflammatory factors (Simons et al. 2021).

There is a paucity in the studies undertaking SHBG gene expression in livestock especially water buffaloes. Therefore, the present study was undertaken on the Egyptian buffaloes (*Bubalus bubalis*) to: 1- characterize the concentrations of testosterone and SHBG in the blood, 2- evaluate SHBG transcription in the liver, 3- sequence specific regions in the SHBG gene, 4- explore whether the mutations, if any, affect the expression of SHBG and testosterone quantities in the buffalo’s circulation, and 5- investigate whether there is a relation between the levels of SHBG transcripts and testosterone and SHBG protein quantities in the blood.

## Materials and methods

### Animals

This study was conducted on 85 Egyptian buffaloes slaughtered in the local abattoirs of Damietta Governorate, including 55 males and 30 females. Males were divided into 4 age-groups (30, 32, 36, and 42 months) while females constituted one group (54 months). Animals were grouped based on the findings of the previous studies that the physiological stages of puberty and sexual maturity in male buffaloes are characterized by distinct hormonal changes. Buffaloes attain puberty between 16 and 40 months, but the average time is over 30 months of age (Sharma et al. 1984), and the sexual maturity occurs after 30 months; at 30 to 33 months of age (McCool and Entwistle 1989).

### Blood and liver tissues sampling

Blood samples were collected in clean and dry tubes without anticoagulants. After incubation for 1 h at 25 °C, sera were collected by centrifuging samples for 10 min at 2500×g. 50–100 mg pieces of liver tissues were collected during the period from March 2019 to December 2020. Aliquots of blood samples were kept at ‒20 °C for further SHBG and testosterone assays by Enzyme-linked immunosorbent assay (ELISA). Liver specimens were ground in liquid nitrogen and immediately resuspended in easy-RED reagent (#17063, iNtRON Biotechnology, South Korea) for RNA extraction and DNA sequencing. Hormonal assays concentration of total testosterone in serum was estimated by ELISA kit (#DK.040.01.3, Abia, Germany). The intra- and inter-assay coefficients of variation are 3.2% and 2.4%, respectively. The sensitivity of assay is 0.2 nmol/l. Level of serum SHBG was measured using bovine ELISA kit (#E-0095BO, Cloud-Clone Crop., USA). Optical density was recorded at the recommended wavelength (450 nm) by a microtiter plate reader within 15–20 min. Unknown testosterone concentrations were obtained from a standard curve by interpolation.

### RNA extraction and quantitative real-time polymerase chain rection (qRT-PCR)

Whole RNA was isolated from liver specimens of buffaloes using easy-RED solution (#17063, iNtRON Biotechnology, South Korea). Complementary deoxyribonucleic acid (cDNA) was made using RevertAid First Strand cDNA Synthesis kit (#K1622, Thermo Scientific, USA) following the provider’s recommendations. QRT-PCR was done on CFX96 Real Time PCR detection system (BIO-RAD, USA) using SYBR Green Master Mix (#K0251, Thermo Scientific, USA) and the primers mentioned in Table 1. As recommended with SYBR Green mix, 25 µl-scale reactions were cycled as follows: 95 °C for 10 min (initial denaturation) and 35 cycles of 95 °C×15 s (denaturation), 60 °C×30 s (annealing), and 72 °C×30 s (extension). Target gene expression was standardized to β-actin expression. Data were analyzed by the ΔCt approach, and SHBG expression fold changes were calculated.

**Table 1:** Primers used in PCR and sequencing

Name	Sequence (5′ to 3’)	Use	Primer position
SH1_F	GGACGAAGTGGTCCTCACTG	Amplification and sequence	‒1043 to ‒1024
SH3_R3	CTGTCCCACTCCTGCACCTG	Amplification and sequence	178–197
SH3_R2	CTCCAATGACTCCGGGGCAA	Sequence	29–48
SH8_F2	TTCCCTCCATGACCAGCTCT	Amplification and sequence	2441–2460
SH11_R2	TCCCTCCCAGAGGTTTCCTT	Amplification and sequence	3365–3384
SH11_R3	TTCTTACCTGGCAAAGCCCC	Sequence	3262–3281

### PCR and DNA sequencing

Based on serum levels of SHBG, 24 male samples representing different-age buffaloes and 6 female samples were selected for DNA extraction. DNA was extracted by the standard ethanol precipitation protocol from the organic phase of the liver samples resuspended in easy-RED solution. PCR was performed on isolated DNA to amplify 1240 bp including 1043 bp from the promoter region and 197 bp from coding sequence of SHBG gene by the primer pair SH1_F/SH3_R3 (Table 1). The middle region of SHBG gene (944 bp spanning the positions 2441 to 3384) was amplified by the primer pair SH8_F2/SH11_R2 (Table 1). All reactions were performed in 50 µl scale. 25 µl of Platinum™ Hot Start PCR master mix (#13000-013, Invitrogen, USA), 2 µl of the forward primer, 2 µl of the reverse primer, and 3 µl of DNA sample (~ 500 ng) were put together in individual PCR mixtures, and the reactions were completed with nuclease-free water to 50 µl. Cycling reactions was as follows: 94 °C×2 min (initial enzyme activation) and 35 cycles of 94 °C×30 s (denaturation), optimized temperature×30 s (annealing), and 72 °C×1–2.5 min (extension). Reactions were ended at 72 °C×10 min (final extension). PCR amplicons were viewed on agarose gel (1.5-2%). Amplicons were cleaned by MEGAquick-spin™ Plus fragment DNA purification kit (#17290, iNtRON Biotechnology, South Korea). DNA sequencing was achieved in the sequencing facility of Macrogen (Seoul, South Korea) using the respective oligonucleotides (Table 1).

### Statistical analysis

All data were statistically checked by STATISTICA program (ver. 12). The means of testosterone, SHBG mRNA, and SHBG protein levels between more than 2 groups were compared by 1-way ANOVA followed by post hoc LSD test. The Correlations of age, testosterone, SHBG expression, and SHBG protein were analyzed using Spearman´s correlation coefficient test in R package and the results were visualized by the R psych package (Revelle 2017). Comparison of the means of 2 groups was done by independent samples t-test. Differences between means were considered significant if *P* ≤ 0.05. Genotype frequencies for the studied population were checked for Hardy-Weinberg equilibrium (HWE) by Chi-square (χ2) test. Statistical graphs were generated by SigmaPlot 15.0 statistical software (SigmaPlot Software Inc., Germany).

## Results

### Serum testosterone concentration

**Fig. 1:**
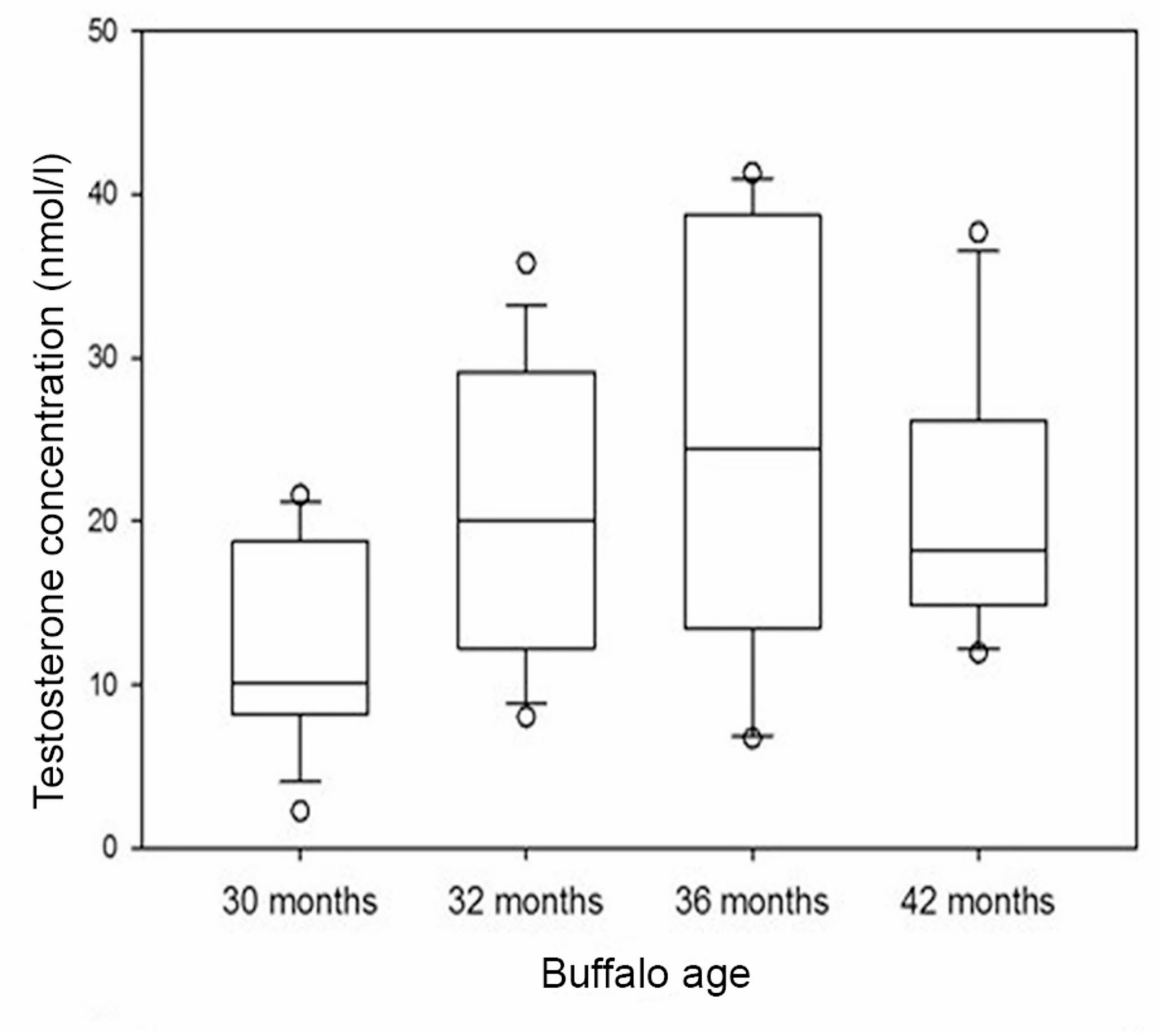
Plot box representing serum testosterone concentration (nmol/l) in buffalo males at 30, 32, 36 and 42 months of age

Serum testosterone concentration in male buffalo increased significantly with age increase from 30 to 32 months (*P* = 0.034) to 36 months (*P* = 0.002) and then declined slightly at 42 months of age (*P* = 0.372) (Fig. 1).

### SHBG mRNA levels

**Fig. 2:**
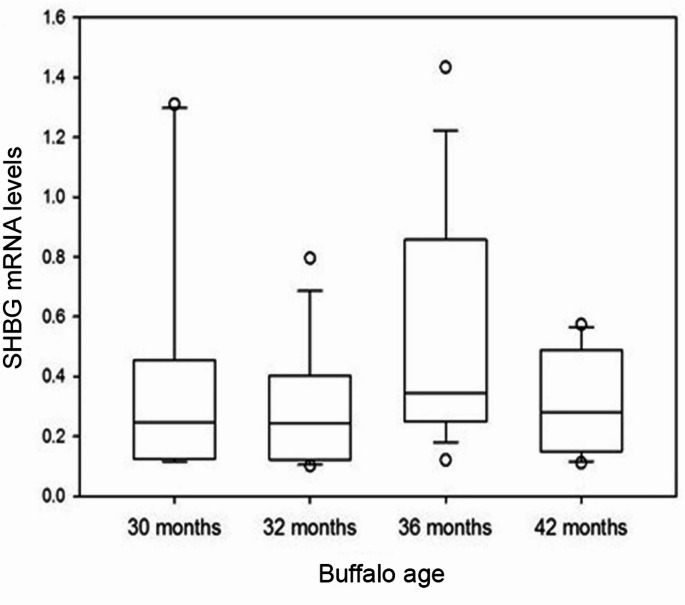
Plot box representing SHBG mRNA levels in livers of male buffaloes at 30, 32, 36 and 42 months of age

SHBG mRNA levels in the hepatic cells of male buffaloes presented some, but non-significant, changes during the different ages. From 30 to 32 months (*P* = 0.38), to 36 months (*P* = 0.08) and at 42 months (*P* = 0.11), levels of SHBG mRNA fluctuated slightly without statistical significance (Fig. 2).

### Serum SHBG protein levels

**Fig. 3:**
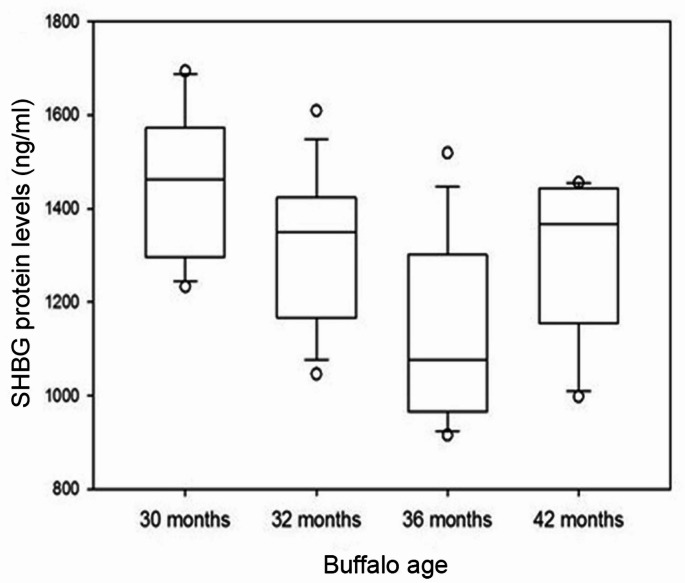
Plot box representing serum SHBG protein levels (ng/ml) in male buffaloes at 30, 32, 36 and 42 months of age

In the male buffalo groups, the serum levels of SHBG protein declined significantly from 30 month-male buffaloes through the 32 month-male buffaloes (*P* = 0.025) to the 36 month-male buffaloes (*P* = 0.011) and then increased slightly again at 42 months of age (*P* = 0.032) (Fig. 3).

### Relation between age, testosterone concentration, and SHBG mRNA expression

**Fig. 4:**
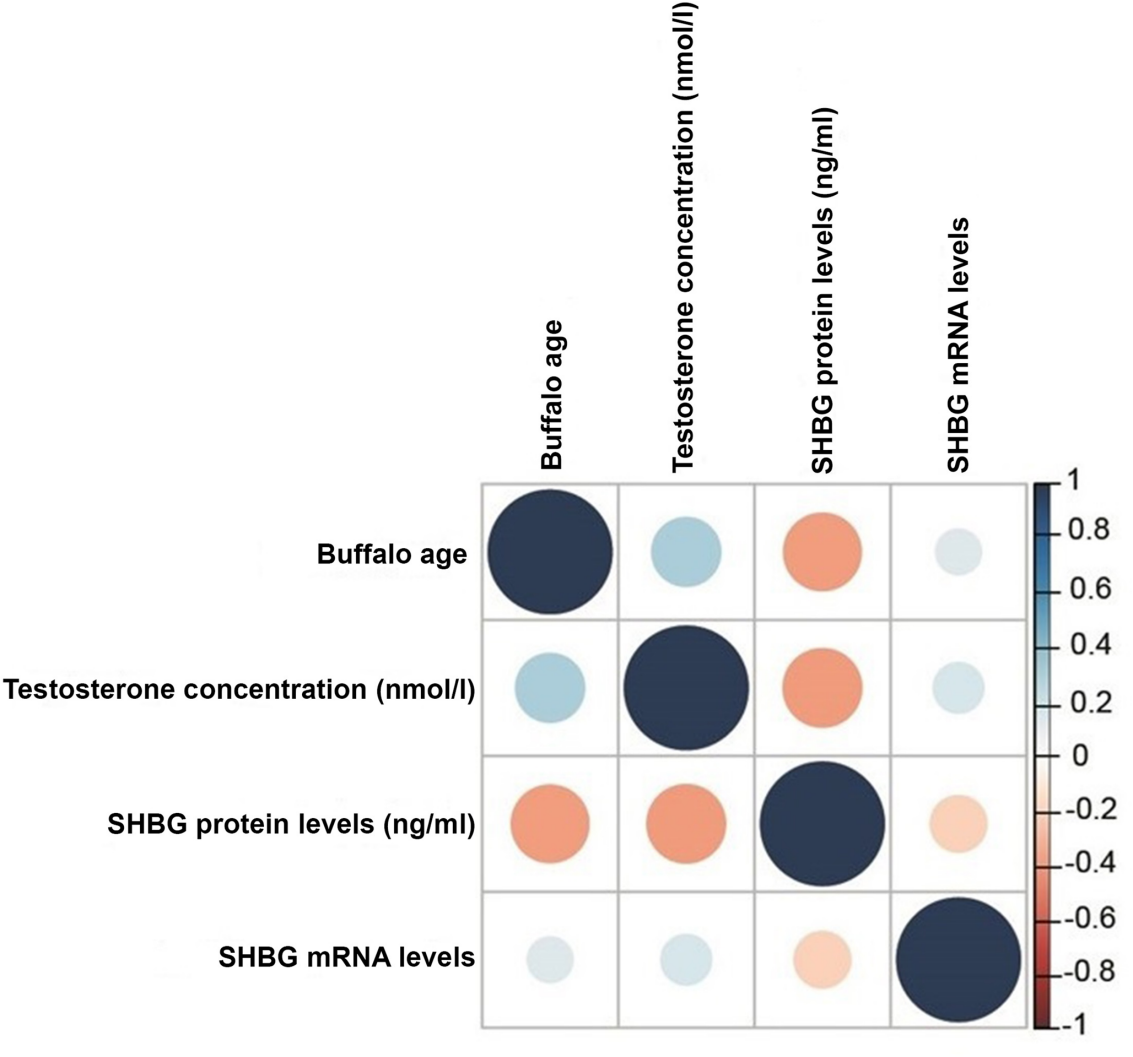
Spearman correlations between buffalo age, testosterone concentration, SHBG mRNA levels, and SHBG protein levels

The relation between age, testosterone concentration, SHBG mRNA level and SHBG protein level in male buffaloes were investigated by Spearman’s correlation coefficient test. Serum testosterone concentration was in a significant positive correlation with male buffaloes’ age (r_s_ = 0.308, *P* = 0.02) (Fig. 4). However, SHBG protein level was in a significant negative correlation with male buffaloes’ age (r_s_ = -0.390, *P* = 0.003) (Fig. 4). SHBG mRNA was in a non-significant positive correlation with male buffaloes’ age (r_s_ = 0.134, *P* = 0.349) (Fig. 4). Serum testosterone concentration was in a significant negative correlation with SHBG protein level (r_s_ = -0.399, *P* = 0.003) (Fig. 4) and a non-significant positive correlation with SHBG mRNA level (r_s_ = 0.144, *P* = 0.314) (Fig. 4). Finally, SHBG mRNA level was negatively but non-significantly related to SHBG protein concentration (r_s_ = -0.173, *P* = 0.226) (Fig. 4).

### Genetic diversity of buffalo SHBG gene promoter

Thirty DNA samples representing different-age groups of Egyptian buffaloes were subjected to amplification and sequencing of around 1 Kbp of SHBG gene promoter and the obtained sequences were compared to the predicted sequence of SHBG promoter in *Bubalus bubalis* isolate 160,015,118,507, breed “Murrah” (NCBI Refseq NC_059159.1). This RefSeq record is a part of a whole-genome shotgun assembly released by the National Dairy Development Board, India. Our sequence results were compared to the reference sequence and the data were summarized in a nucleotide variance report (Table S1). As shown in this report, 1 or 2 variant sites were detected in 19 buffalo DNA samples. Depending on the promoter nucleotide variance, 6 genotypes out of 9 possible genotypes were found (Table S1). Chi-Square analysis showed that the 2 SNPs (‒703G/A and ‒674 C/T) detected in the studied buffalo population were in Hardy–Weinberg equilibrium (χ2 = 1.51 and 0.068, *P* = 0.219 and 0.794, respectively). The relevant sequences were uploaded to the GenBank data source with the accession numbers PP763401‒ PP763406.

Compared to the means of SHBG mRNA level within the same buffalo age group (Table 2), buffaloes with genotypes 1 (GG-CC), 2 (GA-CC), 4 (GG-CT) and 6 (GA-CT) didn’t seem to have a distinctive level of SHBG mRNA in the liver cells of buffaloes; however buffaloes with genotypes 3 (GA-TT) and 5 (GG-TT) had higher levels of SHBG mRNA (Table 2), pointing to a potential association of the allele ‒674T with increased transcription of SHBG gene.

**Table 2:** The relation between SHBG mRNA levels and 6 different genotypes for the ‒703 and ‒674 positions of SHBG promoter in Buffalo DNA samples

Buffalo No.	56	78	87	25	94	99	45	48	60	41	66	73	90	116	97	108	110	92	103	113	51	84	85	121	148	149	139	143	163
Buffalo age	30 months						32 months						36 months					42 months						54 months					
*Mean of SHBG mRNA level	0.3	0.4	0.4	0.3	0.4	0.4	0.3	0.3	0.3	0.3	0.3	0.3	0.5	0.5	0.5	0.5	0.5	0.3	0.3	0.3	0.3	0.3	0.3	0.3	0.3	0.29	0.3	0.29	0.3
Sample SHBG mRNA level	1.3	0.4	0.1	1.3	0.5	0.2	0.1	0.1	0.8	0.1	0.5	0.2	0.9	0.9	0.2	0.3	0.4	0.6	0.2	0.3	0.5	0.3	0.4	0.2	0.6	0.3	0.2	0.3	0.1
Genotype	2	6	1	1	2	1	6	1	2	4	5	2	4	1	1	1	1	1	4	6	6	4	4	4	3	5	6	1	1

*Mean of SHBG mRNA levels does not include the sample under comparison; for example, when the sample No. 56 is compared, the mean of SHBG mRNA level refers to the mean of all samples except of sample No. 56. SHBG mRNA level refers to the fold change of SHBG mRNA relative to the reference gene

### Genetic variations of the middle region of buffalo SHBG gene

The same 30 DNA samples studied for the genetic diversity of SHBG gene promoter were subjected to sequencing 944 bp of the middle region of SHBG gene. The resultant sequences (773 bp long) were compared to SHBG gene sequence in Bubalus bubalis reference (NC_059159.1) and the data were summarized in a nucleotide variance report (Table 3). As shown in this report, 3–10 polymorphic sites were detected in each of these 30 DNA samples. Depending on the occurrence of these polymorphisms, the studied buffaloes were categorized into 16 genotypes (P1-P16) (Table 3). The sequences of these genotypes were uploaded to the GenBank data resource with the accession numbers PP818828 ‒ PP818843. Analysis of allele frequencies and genotype distribution of these polymorphisms by Chi-Square tests indicated that 16 SNPs in the studied buffalo population were in Hardy–Weinberg equilibrium (χ2 = 0.009‒1.95, *P* = 0.163‒0.926, respectively).

**Table 3:** Nucleotide variance report of the middle region of SHBG gene in thirty Buffalo samples

SHBG gene Ref. Seq.		Buffalo No.																													
		25	41	45	48	51	56	60	66	73	78	84	85	87	90	92	94	97	99	103	108	110	113	116	118	121	139	143	148	149	163
2552	C					Y		Y																	Y						
2618	C																													Y	
2648	C		Y						Y		Y	Y																		Y	
2717	T					W		W		W							W														
2720	G			R		R			R				R		R					R			R		R	R	R		A		
2787	A		R	R		R			G		R	G	R		R					R			R		R	R	R		G	G	
2825	A																R														
2844	A					R			G				R		R					R			R		R	R	R		G	G	
2850	A																													R	
2897	C		S	S		S			G		S		S		S					S			S		S	S	S		G	G	
3005	G									R																					
3066	G																R														
3076	C		Y	Y		Y			T		Y	T	Y		Y					Y			Y		Y	Y	Y		T	T	
3089	C	T	Y	Y	T	Y	T	T		T	Y		Y	T	Y	T	T	T	T	Y	T	T	Y	T	Y	Y	Y	T			T
3093	G	A	A	R	A	R	A	A		A	R	A	R	A	A	A	A	A	A	R	A	A	R	A	R	R	R	A			A
3154	A	G	G	R	G	G	G	G	R	G	G	G	R	G	R	G	G	G	G	G	G	G	G	G	R	R	G	G		G	G
**Genotype**		**P1**	**P7**	**P8**	**P1**	**P16**	**P1**	**P2**	**P9**	**P3**	**P10**	**P4**	**P12**	**P1**	**P11**	**P1**	**P6**	**P1**	**P1**	**P13**	**P1**	**P1**	**P13**	**P1**	**P15**	**P12**	**P13**	**P1**	**P5**	**P14**	**P1**

S = (C or G), W = (A or T), R = (A or G), Y = (C or T)

Compared to the means of testosterone and SHBG protein levels in the buffaloes grouped in the same age (Table S2), individual buffaloes with genotypes P4, P8, P9, and P16 had decreased concentration of serum testosterone and increased levels of serum SHBG protein. Buffaloes with genotypes P2, P5, P10, and P11, however, had increased serum testosterone concentration and decreased SHBG protein levels. Buffaloes with genotypes P3, P6, and P7 had higher serum testosterone concentration and SHBG protein levels, while buffaloes with genotypes P14 and P15 had decreased concentrations of serum testosterone and SHBG protein (Table S2).

## Discussion

Serum testosterone concentration in male Egyptian buffaloes was low at 30 months of age, increased significantly through 32 months to a peak level at 36 months, and slightly declined at 42 months. These results back our previous observations (Naeem et al. 2018), where a comparatively low serum testosterone concentration was observed in 12-month and 18-month buffalo bulls, and a significant rise was observed in 24-36-month bulls. In Egyptian buffalo bull calves, the first significant increase in serum testosterone concentration occurred at the age 8–9 months through 13–15 months and a sharp rise was recorded at 17–19-month bulls (Hemeida et al. 1985). In the same context, Elnagar et al. (2022) found that plasma testosterone concentration in Egyptian buffalo males increased from 6 to 12 through 12–18 and 18–24 months of age intervals.

In Pakistani Nili Ravi buffalo bulls, a significant serum testosterone concentration upsurge was observed at 25 months, peaking at 38 month-bulls (Ahmad et al. 1984). In Indian buffalo males, there was an outstanding serum testosterone concentration increase at 24–30 months up to 42 months, with a sharp increase at 48 months (Sharma et al. 1984). Serum testosterone concentrations in Indian buffalo males were very low at 1–24 months, and an increase was detected at 3.5–4 years (Gulia et al. 2010). The above-mentioned observations show a rise in serum testosterone concentration that is directly associated with the buffalo age as a consequence of testes growth and cellular differentiation with age increase (Sharma et al. 1984; Gulia et al. 2010) with slight fluctuations in the different studies owing to the differences in breed, climate, diets, and age (Gulia et al. 2010; Qadarsina et al. 2019; Affandhy et al. 2018). The sensitivity and accuracy of measurement methods may also contribute to such slight fluctuations (Pineda and Dooley 2003). The current study results indicated a positive correlation of testosterone concentration with the age of male buffaloes. An analogous conclusion was reported by Qadarsina et al. (2019) where a highly positive correlation of serum testosterone concentration with age in Simeulue buffalo was detected. Moreover, Gangadhar Jadhav et al. (2018) discovered a positive correlation in Murrah buffalo, while Ammar et al. (2021) documented a low correlation in Gayo buffalo.

The 30 month-male buffaloes had a high level of serum SHBG that declined through 32 to 36 months, followed by an increase at 42 months. Up to a recent review of literature, no previous studies addressed SHBG levels in *B. bubalis* buffaloes. Our results are supported by some previous reports on SHBG in humans and other animals. Hodges et al. (1983) reported significantly higher levels of SHBG in immature males of marmosets than in mature males. In rabbits, SHBG levels surged from 3-day age to reach the highest level at 4-week age, followed by a rapid decrease at 15 week-age (Wong et al. 2001). According to Elmlinger et al. (2002)d rensen et al. (2007), boys aged 6‒8 years had higher levels of SHBG than girls and then SHBG levels declined with the increase of age in both sexes and adult levels were reached at 16–18 years of age with the lowest SHBG levels in boys. Sørensen et al. (2007) attributed this decline in SHBG levels to the rising androgen levels in boys exerting additional negative impact on SHBG production. In the current study, serum SHBG protein level negatively correlated with male buffaloes’ age and with serum testosterone concentration. In line with our data, Hodges et al. (1983) recorded a negative correlation between concentrations of SHBG and testosterone in marmosets. SHBG protein level was significantly and negatively correlated with serum testosterone concentration in boys (Sørensen et al. 2007). A negative correlation between serum SHBG levels and men’s age was also reported (Parwanto 2017; Luo et al. 2018).

The expression of buffalo SHBG gene at the mRNA level was slightly high at 30 months then declined at 32 months. This was followed by a marked rise at 36 months and a second decline at 42 months. This fluctuation in SHBG mRNA levels through different age groups was statistically non-significant. Up to our recent information, the current study evaluates, for the first time, SHBG expression at both mRNA and protein concentrations in Egyptian buffaloes. According to Wong et al. (2001), in rabbit liver cells, SHBG mRNA expression increased from 3 day-age to 10 week-age, a gradual decline thereafter. Li et al. (2015) proposed an association of SHBG mRNA quantities with age. In the hepatic cells of Sprague–Dawley male rats, these authors found that SHBG mRNA levels were lowest in newborn rats, increased through 2 to 6 months, and attained the highest level at 12 months. Our results indicated a weak positive correlation between SHBG mRNA quantity and both male buffaloes’ age and serum testosterone concentration, whereas a weak negative correlation was observed with SHBG protein levels. By regression analysis, Winters et al. (2014) found that SHBG mRNA quantities upsurged with the age in women but not men. Also, these authors documented a non-significant positive correlation between SHBG mRNA and serum testosterone concentration, among men and a significant positive correlation with serum SHBG protein in both genders.

Our study on the variance of SHBG gene promoter revealed the existence of 2 polymorphic positions (‒703 and ‒674) and that the 2 respective genotypes (GG‒TT) and (GA‒TT) present increased levels of SHBG mRNA in the liver cells, suggesting a potential association with increased SHBG transcription. Up to recent literature investigation, no study addressed the relationship between SHBG promoter variance, the quantities of serum testosterone, and SHBG expression in buffaloes. For human, several studies concluded that the circulating quantities of human SHBG protein and testosterone are modulated by inherited causes (Ahn et al. 2009; Ohlsson et al. 2011; Chen et al. 2013; Sung and Song 2016). Through binding to a nuclear factor, the (TAAAA)n repeat polymorphism (rs5030991) situated ~ 800 bp ahead of the transcriptional start site modulated the transcription of human SHBG gene (Cousin et al. 2004). Xita et al. (2011) concluded that men homozygous for the allele (TAAAA)_6_ had lower levels of SHBG than heterozygous or non-carriers of this allele, while Turk et al. (2008) found significantly higher levels of serum SHBG in men homozygous for the same allele than men with other genotypes. Hogeveen et al. (2001) discovered that a 46‒kD nuclear factor favorably interacts with this 6-repeat allele and the downstream elements of the SHBG promoter to modulate the transcriptional activity of SHBG promoter. According to Ahn et al. (2009), the G/A SNP (rs1799941) that lies 67 bp ahead of the transcriptional start site of human SHBG gene was strongly associated with increased serum testosterone concentration in men. Moreover, women and young non-diabetic obese males with AA genotype had higher SHBG protein levels than those with GG or GA genotype (Riancho et al. 2008; Sunbul et al. 2013; Castellano-Castillo et al. 2019), Similarly, men carrying the minor homozygote genotype have increased serum testosterone and SHBG protein quantities compared with those with the major homozygote genotype (Svartberg et al. 2014).The concluded relationship between the SNP rs1799941 and blood SHBG proposes a leading role in modulating blood SHBG levels (Wickham et al. 2011).

Some studies demonstrated that the SHBG production is regulated by changes in the hepatic levels of HNF4α plus COUP-TF1. HNF4α and COUP-TF1 compete for the same DNA-binding site spanning the nucleotides − 30 to -26 and act as on-off switch for SHBG transcription (Jänne and Hammond 1998). HNF4α binding promotes transcription activity, while COUP-TF1 suppresses it (Jänne and Hammond 1998). Thyroid hormone is thought to indirectly up-regulate the manufacturing of plasma SHBG through enhancing HNF4α levels in the hepatic cells (Selva and Hammond 2009b). In contrast, PPAR-2γ down-regulates hepatic SHBG expression in vitro by binding to the DR-1 element in the proximal 299 bp region of the human SHBG promoter (Selva and Hammond 2009a); specifically, the nucleotides − 88 to -66 before the transcriptional start site (Jänne and Hammond 1998).

In an earlier investigation (Naeem et al. 2018), we sequenced the full length of SHBG gene in *Bubalus bubalis* bulls and noticed the presence of many nucleotide variations in the middle region of SHBG gene. In the present study, we further dissected the nucleotide variance of this region (773 bp) in 30 buffaloes. Our results determined a 603 bp-region spanning the nucleotides 2554–3154 as a highly polymorphic. Obviously, this study revealed that the alleles 3089T, 3093 A, and 3154G are the major alleles but not the corresponding alleles in the reference SHBG sequence. In addition, our results indicated that four buffalo genotypes showed a decreased concentration of serum testosterone and an increased level of serum SHBG protein, while four genotypes showed an increased concentration of serum testosterone and decreased level of serum SHBG protein. Three genotypes showed increased concentrations of both testosterone and SHBG protein, while two genotypes showed decreased concentrations of both testosterone and SHBG protein.

NCBI database predicted 6 transcript variants (X1–X6) of *B. bubalis* SHBG gene, the longest of which is X1 (XM_006062863.4) and the coding sequence is composed of 8 exons. The polymorphic region sequenced in this study spans most of exon 6, the entire intron 6, and half of exon 7 and encodes 71 amino acids within this transcript (amino acids 266–336). The current data supports our previous suggestions (Naeem et al. 2018). Furthermore, the possible importance of the substitutions in this region is supported by some other reports on human SHBG protein. The amino acid substitution E326K (produced by a SNP in exon 8 of human SHBG gene) was associated significantly with decreased levels of serum SHBG in polycystic ovary syndrome women having an increased copy number of the allele containing this substitution (Hacıhanefioğlu et al. 2013). Men who carry the N327 allele of SHBG gene had higher concentrations of testosterone and SHBG protein (Vanbillemont et al. 2009). Similarly, hirsute women with substitution D327N (SNP 6259) have significantly higher SHBG protein levels (Cousin et al. 2004). This polymorphism is located in the 8th exon of men SHBG gene, and it introduces an extra site for N-glycosylation (Power et al. 1992). In SHBG protein with the N327 allele, the additional carbohydrate chain reduces its metabolic clearance rate. This allele is thought to be linked to an improved half-life of the protein and, consequently, high levels of SHBG protein (Cousin et al. 1998).

Men with variant allele (CC or CT) of SNP rs6257 (located 17 bp upstream of exon 2) were found to have lower concentrations of plasma SHBG (Ding et al. 2009; Eltarhouny et al. 2015) and testosterone (Eltarhouny et al. 2015) than homozygote men for the wild-type allele (TT) (Power et al. 1992; Cousin et al. 1998). Ding et al. (2009) concluded that the associations with the SNP rs6257 suggest the existence of a likely important splicing or regulatory element in this area.

## Conclusion

In conclusion, in the endeavor to find a reliable way for selecting *B. bubalis* bulls with high serum testosterone concentration, which are highly demanded for breeding, the current study characterized serum testosterone concentration in 4 different-age bulls and one female buffalo group in Egypt, and tried to dissect the relation between serum testosterone concentration, SHBG gene transcription level, and SHBG protein concentration in the buffaloes’ blood. Furthermore, the study investigated the genetic variation of both the SHBG gene promoter and the polymorphic region in the middle of SHBG gene in 30 buffaloes representing the different-age groups under study. Based on the relation between these genetic variations and the concentrations of serum SHBG protein and testosterone, the study suggested potential associations between different genotypes and high or low serum testosterone concentration. We believe that extended characterization of a limited number of potential SNPs may lead to more definite conclusions. The feasible and successful selection of *B. bubalis* bulls with high serum testosterone concentration is of interest to animal breeders and the livestock economy in general.

## Supplementary Information

Below is the link to the electronic supplementary material.

Supplementary Material 1

Supplementary Material 2

## Data Availability

Research datasets are available at Mendeley Data repository (https://data.mendeley.com/datasets/n9bnhbr3g9/2). Sequence data are deposited in GenBank data resource under the accession numbers (PP763401 ‒ PP763406 and PP818828 ‒ PP818843). Any other data can be requested from the corresponding author.
