# Supplementary material for: Expression of sex hormone-binding globulin gene and its relation to serum testosterone concentration in Bubalus buffaloes
Source: Trop Anim Health Prod. 2025 Aug 5;57(7):343. doi: 10.1007/s11250-025-04592-4 (PMC12325477; doi:10.1007/s11250-025-04592-4)
Supplement: Supplementary file 1 — Supplementary Material 1 [file 11250_2025_4592_MOESM1_ESM.doc]

**Table S1** Nucleotide variance report of the promoter region of SHBG gene in thirty buffalo samples

| **SHBG gene**  **Ref. Seq.** | | **Buffalo No.** | | | | | | | | | | | | | | | | | | | | | | | | | | | | | |
| --- | --- | --- | --- | --- | --- | --- | --- | --- | --- | --- | --- | --- | --- | --- | --- | --- | --- | --- | --- | --- | --- | --- | --- | --- | --- | --- | --- | --- | --- | --- | --- |
|  |  | **25** | **41** | **45** | **48** | **51** | **56** | **60** | **66** | **73** | **78** | **84** | **85** | **87** | **90** | **92** | **94** | **97** | **99** | **103** | **108** | **110** | **113** | **116** | **118** | **121** | **139** | **143** | **148** | **149** | **163** |
| **-703** | **G** | GG | GG | GA | GG | GA | GA | GA | GG | GA | GA | GG | GG | GG | GG | GG | GA | GG | GG | GG | GG | GG | GA | GG | GA | GG | GA | GG | GA | GG | GG |
| **-674** | **C** | CC | CT | CT | CC | CT | CC | CC | TT | CC | CT | CT | CT | CC | CT | CC | CC | CC | CC | CT | CC | CC | CT | CC | CT | CT | CT | CC | TT | TT | CC |
| **Genotype** | | 1 | 4 | 6 | 1 | 6 | 2 | 2 | 5 | 2 | 6 | 4 | 4 | 1 | 4 | 1 | 2 | 1 | 1 | 4 | 1 | 1 | 6 | 1 | 6 | 4 | 6 | 1 | 3 | 5 | 1 |
