# Supplementary material for: Expression of sex hormone-binding globulin gene and its relation to serum testosterone concentration in Bubalus buffaloes
Source: Trop Anim Health Prod. 2025 Aug 5;57(7):343. doi: 10.1007/s11250-025-04592-4 (PMC12325477; doi:10.1007/s11250-025-04592-4)
Supplement: Supplementary file 2 — Supplementary Material 2 [file 11250_2025_4592_MOESM2_ESM.doc]

**Table S2** The relation between serum testosterone and SHBG protein levels, and different buffalo genotypes based on the SNPs in the promoter and middle region of SHBG gene in thirty buffalo samples

| **Buffalo No.** | **56** | **78** | **87** | **25** | **94** | **99** | **45** | **48** | **60** | **41** | **66** | **73** | **90** | **116** | **118** | **97** | **108** | **110** | **92** | **103** | **113** | **51** | **84** | **85** | **121** | **148** | **149** | **139** | **143** | **163** |
| --- | --- | --- | --- | --- | --- | --- | --- | --- | --- | --- | --- | --- | --- | --- | --- | --- | --- | --- | --- | --- | --- | --- | --- | --- | --- | --- | --- | --- | --- | --- |
| **Buffalo age** | **30 months** | | | | | | **32 months** | | | | | | **36 months** | | | | | | **42 months** | | | | | | **54 months** | | | | | |
| ***Mean of testosterone levels (nmol/l)** | 12.6 | 12.1 | 13.1 | 13 | 12.1 | 12.9 | 21.6 | 21.3 | 19.6 | 20.2 | 21.1 | 20.6 | 24.3 | 23.2 | 23.2 | 24.3 | 25.4 | 24.2 | 20.4 | 19.2 | 21.7 | 20.7 | 21.4 | 20.4 | 2.1 | 2.2 | 2.2 | 2.2 | 2.2 | 2.1 |
| **Sample testosterone level (nmol/l)** | 14.5 | 20.6 | 8 | 8.9 | 19.9 | 10.1 | 9.7 | 13.9 | 35.8 | 27.6 | 14.9 | 22.8 | 24.4 | 41.3 | 2.7 | 23.5 | 6.9 | 25.5 | 26.2 | 37.7 | 13.2 | 5.3 | 16.5 | 26.2 | 5.9 | 2.8 | 0.7 | 1.1 | 1.6 | 4.3 |
| ***Mean of SHBG protein level (ng/ml)** | 1475 | 1478 | 1459 | 1449 | 1439 | 1450 | 1304 | 1322 | 1316 | 1303 | 1292 | 1303 | 1156 | 1157 | 1156 | 1126 | 1125 | 1119 | 1302 | 1319 | 1303 | 1282 | 1273 | 1302 | 1313 | 1315 | 1311 | 1295 | 1298 | 1293 |
| **Sample SHBG protein level (ng/ml)** | 1272 | 1234 | 1455 | 1577 | 1694 | 1569 | 1344 | 1108 | 1183 | 1357 | 1488 | 1356 | 927 | 916 | 939 | 1412 | 1429 | 1519 | 1159 | 998 | 1154 | 1368 | 1452 | 1421 | 1158 | 1099 | 1225 | 1683 | 1600 | 1735 |
| **Genotype**  **(-703)** | GA | GA | GG | GG | GA | GG | GA | GG | GA | GG | GG | GA | GG | GG | GA | GG | GG | GG | GG | GG | GA | GA | GG | GG | GG | GA | GG | GA | GG | GG |
| **Genotype**  **(-674)** | CC | CT | CC | CC | CC | CC | CT | CC | CC | CT | TT | CC | CT | CC | CT | CC | CC | CC | CC | CT | CT | CT | CT | CT | CT | TT | TT | CT | CC | CC |
| **Genotype no.**  **(Middle region)** | P1 | P10 | P1 | P1 | P6 | P1 | P8 | P1 | P2 | P7 | P9 | P3 | P11 | P1 | P15 | P1 | P1 | P1 | P1 | P13 | P13 | P16 | P4 | P12 | P12 | P5 | P14 | P13 | P1 | P1 |

*Mean of testosterone or SHBG protein levels does not include the sample under comparison; for example, when the sample No. 56 is compared, the mean of testosterone or SHBG level refers to the mean of all samples except of sample No. 56.
